# Faith Cures

**Published:** 1883-09-20

**Authors:** 


### *FAITH CURES.*

There seems to be, of late, a growing tendency to accept and believe in the doctrine of Miraculous or Faith Cures. Certain prominent ministers, as the Rev. Mr. Barnes, of Kentucky, and others, have yielded their assent to this idea, and claim to have cured many afflicted people by prayer and the laying on of hands. Dr. Gorton (New York Medical Times) in an article on this subject makes some very sensible remarks touching the influence of the mind on disease, and of faith in the drug or the Doctor as a therapeutic agent. He truly remarks that—

"Its influence is not limited to the domain of religion or emotion. We repeat that Faith is a mental force exerted in the direction of a particular object. It may be claimed to be a leading factor in effecting vital changes in the economy—an indispensable factor, without which many derangements and defects of the organism would remain irremediable. What would become of the marvelous effects attributed to the influence of infinitesimals were it not for the existence of the faith element? As for us, we are more surprised at the alleged effects of minutely attenuated medicines than we are at those of prayer. Some of the cures effected by the high potencies are more marvelous than the so-called miracles of the saint or the religious *devotee*."

"We may not know all the sanative and therapeutic agencies involved in the action of a specific medicine, nor those exerted by a strong-lunged orator in prayer for the same end; but we are in a position to affirm that a *strong belief* in the efficacy of *either*, on the part of both dispenser and recipient, is essential to the production of a beneficial result. Certainly the patient must believe—have faith—in the power of medicine and in the skill of the doctor, and the doctor must likewise have faith in it as well as faith in himself, or the medicine is shorn of the larger part of its sanative virtues and the doctor of his success."

